# Designing and validating an adaptation questionnaire among the husbands of Iranian Muslim women with breast cancer

**DOI:** 10.3389/fpubh.2023.1073032

**Published:** 2023-03-30

**Authors:** Marzieh Beigom Bigdeli Shamloo, Nasrin Elahi, Marziyeh Asadi Zaker, Kourosh Zarea, Armin Zareiyan

**Affiliations:** ^1^Nursing and Midwifery Department, School of Nursing and Midwifery, Ahvaz Jundishapur University of Medical Sciences, Ahvaz, Iran; ^2^Department of Nursing, School of Nursing and Midwifery, Nursing Care Research Center in Chronic Diseases, Ahvaz Jundishapur University of Medical Sciences, Ahvaz, Iran; ^3^Nursing Department, School of Nursing, AJA University of Medical Sciences, Tehran, Iran

**Keywords:** breast cancer, breast neoplasms, adaptation, malignancy, validation, tool

## Abstract

**Background:**

Breast cancer is the most common cancer among women and is the second leading cause of cancer death. This disease affects all aspects of the patient's life and family, especially the patient's spouse, which confirms the need to adapt to these changes. The common instruments used for the investigation of adaptation among husbands of women with breast cancer are mainly outdated, one-dimensional, or non-concordant with the Iranian culture. Therefore, the present study aimed to design and validate an adaptation scale among the husbands of Iranian Muslim women suffering from breast cancer.

**Methods:**

This exploratory sequential mixed study was conducted in two qualitative and quantitative stages. In the qualitative stage, semi-structured interviews were performed with 21 participants. Then, items were developed through content analysis using the approach proposed by Elo and Kyngas on the basis of Roy's adaptation model. In the quantitative stage, the extracted items were reduced and psychometric properties such as face, content, and construct validity as well as reliability were explored. To investigate the construct validity, a cross-sectional descriptive study was conducted on 300 husbands of women with breast cancer selected *via* cluster sampling.

**Results:**

The initial questionnaire contained 79 items. After the assessment of face and content validity, 59 items were evaluated regarding construct validity using exploratory factor analysis. At this stage, six adaptation dimensions were observed among the women's husbands, with the variance of 51.71. The Cronbach's alpha and correlation coefficient of the questionnaire were 0.912 and 0.701, respectively.

**Conclusion:**

The developed 51-item adaptation scale had an appropriate validity and reliability and could be used for the assessment of adaptation in the target group.

## Introduction

Breast cancer (BC) is the most prevalent cancer amongst females all over the world (1). The highest prevalence of BC has been reported in Europe and the United States, while the lowest prevalence has been found among Asian and African women (2). In Iran also, BC accounts for the second cause of cancer-related death (3). In this country, the prevalence of this malignancy has been reported to be 29.88% per 100 000 women (4), mainly occurring in 45–65 and 80–85 age groups (5).

BC can be treated *via* surgery, chemotherapy, and radiotherapy, depending on the severity and stage of the disease (6). In some cases, mastectomy is performed to prevent BC metastasis (7), which involves the partial or complete removal of one or both breasts. This operation leads to the asymmetry of the breasts, a large scar, and change in nipple sensation. In addition, since breasts are orgasm able organs, can secrete oxytocin, and result in uterine contractions during sexual intercourse, their removal may be accompanied by a decline in sexual function (8), eventually exerting negative impacts on patients' mental, sexual, and social statuses (9). Changes in women's sexual behaviors can, in turn, affect their husbands' sexual functions and lead to various psychological consequences (10), which may even result in hospitalization and depression (11). Hence, BC is sometimes referred to as the relational cancer (12).

Support on the part of one's husband plays a key role in adaptation with BC. Supporting women can reduce their stress levels and improve their compatibility (13). It can also improve patients' problems associated with mental imageries, which can avoid depression (14) and promote the quality of sexual relationships (15). On the other hand, incompatible responses on the part of each spouse can expose the couple to serious challenges in their marital relationships (14). They can endanger the family's function, as well (15). One of the prerequisites of supporting one's wife is adaptation with her disease (12). Thus, investigation of adaptation and its dimensions can play a critical role in improving the disease process. In consultation sessions also, adaptation and its dimensions can be taught to patients in order to facilitate their acceptance of the disease, adoption of positive adaptation strategies, and return to normal life (16). In this way, steps can be taken toward the timely diagnosis of mental status among husbands (17).

There are several questionnaires including the Ways of Adaptation Checklist developed by Lazarus and Folkman (18), Tobin's Adaptation Strategies Inventory, Adaptation Inventory for Stressful Situations (19), and Adaptation Strategy Indicator (20), which can be utilized to assess adaptation with stress. Some other questionnaires designed in this context include adaptation with chronic pain (21), sexual pain (22), cancer (23), and family adaptation with eating disorders (23). These questionnaires were developed between 1983 and 2016 and were translated to other languages afterwards. Additionally, these questionnaires are mostly one-dimensional, while cancer can lead to mental, social, and sexual disorders. Hence, it is better to make use of an instrument encompassing all these dimensions (24). Since cultural variables play an important role in adaptation, the intended instrument should contain the inter-personal, cultural, and belief contexts, as well (25). Afterward, In Iran, the religion of Islam and the law allow the husband to remarry if the woman is diagnosed with cancer Or divorce his wife (26), but such permission is not given to Iranian Muslim women to divorce and have sex with another man if the husband is diagnosed, which in some Iranian views is gender discrimination (27). Based on what was mentioned above, the present study aims to design and validate Adaptation Questionnaire among the Husbands of Iranian Muslim Women with Breast Cancer (AQHIMWBC).

## Materials and methods

The methods used in the designing and validating of the questionnaire are based on current best practices (28).

### Designing of items for AQHIMWBC

Firstly, semi-structured questions were asked about the measures taken by the patients' husbands to cope with the condition. Then, using analytical questions, the concept of adaptation was explored *via* content analysis based on Roy's adaptation theory. Callista Roy has introduced adaptation in four dimensions of physiological needs, self-concept, role function, and interdependence (29). Afterwards, the participants' statements were written down by the researcher and coded. Then, the data were analyzed using Elo and Kyngas approach (30).

### Study design and participants

In this exploratory sequential mixed study, the participants who met the inclusion criteria were selected through purposive and convenience sampling. The participants included ten spouses, nine patients, and four therapists who had undergone treatment or worked at Shahid Baghaei treatment center, Ahvaz, Iran. Totally, 23 interviews were conducted with 21 participants. The inclusion criteria of the study were being familiar with Persian language, having appropriate physical and mental states at the time of interview, passage of at least 1 year from marriage, passage of 6 months from the diagnosis of BC, and necessity to undergo mastectomy, chemotherapy, and radiotherapy. The inclusion criteria for the therapists were having at least a BSc degree and having 2 years of work experience with patients suffering from cancer (Table 1).

**Table 1:** The participants' demographic features and underlying information.

**Variable**	**Scale**		**Frequency**
Age (years)	25–35		3 (13%)
	35–45		10 (43%)
	45–55		5 (22%)
	55–65		3 (13%)
Sex	Female		11 (52%)
	Male		10 (48%)
Education level	Below diploma		5 (22%)
	Diploma		7(33%)
	Academic		9 (45%)
Occupation	Self-employed		4 (24%)
	Retired		4 (17%)
	Jobless		7 (20%)
	Employee		6 (20%)
Participant type	Husband		9 (45%)
	Patient		8 (35%)
	Therapist	Nurse	1 (4%)
		Physician	2 (8%)
		Psychologist	1 (4%)
	Patient's mother		1 (4%)
Number of children	1		2 (11%)
	2		16 (76%)
	≥3		3(13%)
Marriage duration (years) (Except for therapists)	1–5		1 (5%)
	5–10		2 (11%)
	10–15		2 (16%)
	15–20		1 (5%)
	>20		11 (63%)
Disease duration (years) (Except for therapists)	< 5		4 (23%)
	5–10		4 (23%)
	>10		9 (54%)
Mastectomy type (Except for therapists)	Partial		5 (29%)
	Total		9 (54%)
	Bilateral		3 (17%)
History of metastasis) (Except for therapists)	Yes		2 (16%)
	No		15 (84%)

### Data collection and management

At first, the participants were informed about the study objectives and procedures as well as the confidentiality of their information. They were also assured that in case of lack of cooperation, they would not be deprived of treatment and no costs would be imposed on them. Then, their informed consent was obtained. Due to the COVID-19 pandemic, all interviews were performed through video call. After introduction of the interviewer and expression of the objectives, the patients' husbands were asked semi-structured questions. It should be noted that the interviews were recorded after gaining the participants' consent. The interviews were begun with questions about adaptation dimensions and interdependent according to Roy's theory and were continued with more specific questions based on the primary interviews and the main themes. Data analysis was manually performed and no software was used for this purpose with Elo and Kyngas approach (30). Then, content analysis was performed based on four dimensions, namely role physical disorder, self-concept, role playing and interdependent. Data analysis was done with MAXQDA version 2020 software.

### Rigor in qualitative stage

Content analysis was done by the researcher who has 12 years of work experience in the field of nursing. In this study, 23 interviews were conducted, which lasted between 25 and 55 min. At the beginning, the researcher took the inclusion criteria into account in order to select the participants. The researcher collected and analyzed the data for a year, so as to determine their trustworthiness. In this context, credibility was confirmed by the participants and experts. Additionally, the transcribed interviews were coded by two researchers and their reliability was approved by the agreement above 0.9. The dependability of the interviews was approved by the restatement of the concepts and their confirmation by the participants. Considering conformability, the methodology was explained in details, so that others would be able to follow up the research processes. Finally, transferability was improved by describing the participants' demographic features.

## Data analysis

The analysis of the quantitative part led to the extraction of 4 dimensions: physical problems, self-concept of role playing and interdependence. In the dimension of physical problems, attention was paid to disorders created in all body systems. In the dimension of self-image, attention was paid to the attitudes, beliefs, spirituality, ideals, mental and physical perceptions of the husbands of women with BC. In the aspect of role playing, attention was also paid to the roles of child, wife, job and all situations that assign tasks to an individual. Also, in the dimension of interdependence, analysis of relationships, all support systems and their aspects, support failures in the husbands of women with BC were mentioned.

After developing the primary items of the scale, its content and face validity as well as its reliability (internal consistency) were assessed.

### Face validity

Initially, the scale included 79 items; 12 in the physiological needs dimension, 22 in the self-concept dimension, 19 in the role function dimension, and 26 in the interdependence dimension. In order to determine the face validity of the instrument, it was given to 12 participants to provide their opinions about the understandability, simplicity, and relevance of the items. Accordingly, 50 items were revised. Considering the quantitative face validity also, the instrument was given to ten faculty members of Ahvaz, Dezful, and AJA universities of medical sciences to provide their answers in form of a five-option Likert scale. After evaluating the item impact, all the questions obtained scores above 1.5 and none of the items was modified.

### Content validity

In order to investigate the qualitative content validity of the scale, some experts were requested to assess the items in terms of grammar, wording, item allocation, and scaling. In order to compute the Content Validity Ratio (CVR), the necessity of the items was assessed by 12 experts using a three-option Likert scale. Based on Lawshe table, the minimum CVR for 12 experts was equal to 0.56. In order to compute the Content Validity Index (CVI) also, the instrument was given to 12 experts. In case the items received scores above 0.79, between 0.7 and 0.79, and below 0.7, they were retained, reviewed, and removed, respectively.

### Construct validity

In order to determine the construct validity of the scale, it was given to 300 husbands of women suffering from BC using a five-option Likert scale. After that, exploratory factor analysis was done *via* the SPSS 22 software. In the first stage of exploratory factor analysis, the prominent items in the factor structure of the scale were identified using a scree plot. It should be noted that factor analysis can be done through a variety of models. Since factor analysis in the present study aimed to summarize the variables and provide a limited number of factors, the Maximum Likelihood (ML) technique was utilized to extract the factors. Then, the factor load matrix was employed to identify the variables related to each factor as well as to make the factors more interpretable. In the obtained matrix, the variables with higher loads on a factor were allocated to that factor. Besides, the variables with factor loads above 0.3 showed an acceptable significance level with their related factors (Table 3).

### Reliability

For determining the internal consistency of the variables and data sufficiency, use was made of the Kaiser-Meyer-Olkin (KMO) test and Bartlett's test of sphericity.

### Ethical approval

The study received approval by the Ethics Committee of the Vice-chancellor for Research Affairs of Ahvaz Jundishapur University of Medical Sciences (ethics code: IR.AJUMS.REC.1399.099). All participants gave their written consent *via* web.

## Results

Based on the results presented in Table 1, this study was conducted on ten husbands, nine patients, four therapists, and one patient's mother. The majority of the participants had academic degrees and had been married for more than 20 years. Additionally, more than 10 years had passed from the diagnosis of BC in most of the participants. The patients had undergone total mastectomy and had no history of metastasis.

The qualitative content analysis revealed four main categories, 18 generic categories, 63 subcategories, and 179 codes. Thus, the initial scale was developed with 79 items. After the assessment of qualitative face validity, 50 items were reviewed and revised. Then, quantitative face validity was evaluated, in which all the items received scores above 1.5 and remained unchanged. After calculating the CVR, 20 items were eliminated due to receiving scores below 0.56. After that, CVI was computed as >0.79 for all the items. Thus, all the 59 items were considered for the assessment of construct validity. Based on the participants' feedbacks as well as the reviews performed by the researcher and the supervisor, items 10, 12, 13, 14, 22, 24, 25, and 52 were removed, and a 51-item scale was developed. Considering KMO = 0.746 and Bartlett's test = 12,398.085 at the significance level of 0.001, the variables were appropriate for factor analysis.

**Figure 1:**
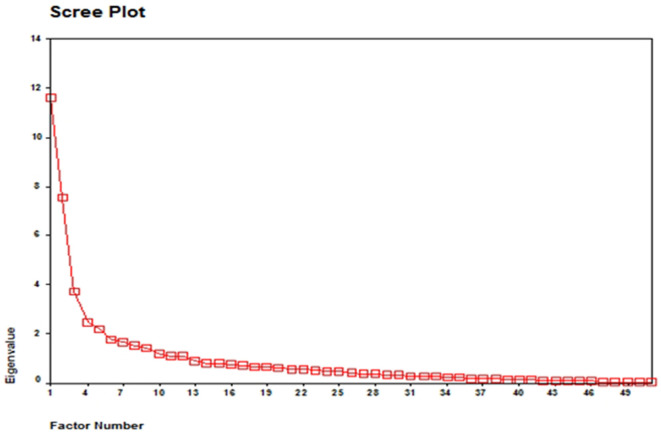
Scree plot of the factor analysis of the adaptation questionaire for the Iranian Muslim husbands of women with BC.

Since six factors obtained eigenvalues > 1, six factors were approved by factor analysis (Figure 1). Based on the results, the six factors explaining a part of the total variance were extracted (Table 2). According to varimax rotation, these six factors explained 51.71% of the changes in the scale variables (Table 3). Furthermore, < 15% of the respondents obtained the highest and lowest possible scores in these six factors, and the scale presented no ceiling or floor effects.

**Table 2:** The extracted factors and their eigenvalues, variance percentages, and cumulative variance percentages.

	**Factors**	**Number of items**	**Eigenvalue**	**Variance percentage**	**Cumulative variance percentage**
1	Role play	17	8.92	17.49	17.49
2	Physical problems	7	4.77	9.37	26.86
3	Mental tensions associated with financial toxicity	12	4.35	8.55	35.41
4	Marital relationships	7	3.27	6.43	41.84
5	Perceived support	4	2.66	5.21	47.06
6	Fear of the future	4	2.37	4.66	51.71

**Table 3:** Rotated factor matrix of AQHIWBC.

**No**.	**Item**	**Factor load**					
		**1**	**2**	**3**	**4**	**5**	**6**
37	I have tried to eliminate my wife's disease complications.	0.832					
35	I accompany my wife during treatment.	0.801					
45	I feel more in love with my wife after her disease.	0.774					
39	During my wife's disease course, I have enhanced my information about cancer control and treatment strategies.	0.773					
47	I sympathize with my patient.	0.763					
44	Due to the disease, we know the value of the moments of our life.	0.734					
38	During my wife's disease course, I have learned stress coping skills.	0.728					
36	During my wife's disease course, I helped her do household chores.	0.723					
26	I feel responsible toward my wife.	0.719					
43	Due to the disease, we pay more attention to our health status.	0.676					
42	We found meaning in our life after this disease.	0.634					
34	During my wife's disease course, training played a positive role in our relationship.	0.617					
48	During the disease course, I improved my in-laws' mood.	0.561					
23	I accepted my wife's disease.	0.453					
20	I had a positive attitude toward the disease.	0.433					
41	I avoid showing pity to my wife.	0.385					
2	I experienced sleep disorder during my wife's disease course.		0.815				
3	I felt weak during my wife's disease course.		0.778				
4	I decreased my physical activities due to my wife's disease.		0.712				
5	I have fluctuating blood pressure due to my wife's disease.		0.672				
6	My heartbeat has increased due to my wife's disease.		0.662				
1	I lost weight due to my wife's disease.		0.608				
7	I had nervous pains like stomachache or hand pain during my wife's disease course.		0.579				
57	I have chosen a second job or I work extra hours to solve my financial problems.			0.753			
59	I feel that I need help due to my wife's disease.			0.746			
58	I feel alone due to my wife's disease.			0.690			
55	I am worried about the costs of treatment.			0.578			
28	There were disruptions in my daily living activities during my wife's disease course.			0.541			
56	I refer to governmental hospitals to reduce the expenses.			0.503			
15	I felt confused in decision-making after being informed about my wife's disease.			0.460			
16	I feel frustrated due to my wife's disease.			0.436			
9	After my wife's disease, I have grown the habit of plucking my hair and biting my fingernails.			0.418			
49	The cost of treatment of my wife is supplied by charities.			0.399			
8	I am tired of my wife's long treatment course.			0.377			
29	I have changed my work plan due to my wife's disease.			0.373			
27	My love for my wife has decreased due to her disease.				0.777		
46	After my wife's disease, the love between us faded away.				0.684		
40	During my wife's disease course, I was indifferent and did not help her.				0.673		
11	I think about divorce.				0.577		
32	I chose another sexual partner after my wife's disease.				0.548		
31	I have changed the type of my sexual relationships.				0.441		
30	I have reduced the frequency of my sexual relationships.				0.423		
50	I am psychologically and financially supported by my family.					0.931	
51	I was supported by my acquaintances during my wife's disease course.					0.708	
53	I received support at my workplace during my wife's disease course.					0.522	
54	The government's support is sufficient for supplying my patient's medications.					0.431	
17	I am worried about the disease and its complications in future.						0.801
18	I am worried about ambiguities in the treatment of my wife's disease.						0.735
21	I am afraid of the disease and its complications.						0.620
19	After I heard about my wife's disease, death came to my mind.						0.524

The Cronbach's alpha coefficient was found to be 0.931, 0.893, 0.855, 0.745, 0.726, and 0.883 for factors 1, 2, 3, 4, 5, and 6, respectively. This measure was also computed as 0.912 for all the factors. Additionally, the split-half correlation coefficient was computed as 0.933 for factor 1, 0.888 for factor 2, 0.834 for factor 3, 0.704 for factor 4, 0.711 for factor 5, 0.852 for factor 6, and 0.701 for the entire factors (Table 4).

**Table 4:** Investigation of reliability; the results of the split-half and internal consistency tests.

**Components**	**Number of items**	**Cronbach's alpha**	**Split-half**	**Within-group correlation**
First	17	0.931	0.933	0.824
Second	7	0.893	0.888	0.799
Third	12	0.855	0.834	0.716
Fourth	7	0.745	0.704	0.643
Fifth	4	0.726	0.711	0.705
Sixth	4	0.883	0.852	0.743
Total	51	0.912	0.701	0.653

## Discussion

The influence of religion on adaptation is very clear (31) and it can affect the compatibility of people from the point of view of the incidence of the disease to its treatment (32). In addition, culture is another effective factor in compatibility, and in cultures where there is gender discrimination, some couples are able to transcend culture and achieve better compatibility (33). Considering that the most common religion in Iran is Islam (34) and the family laws in Iran are such that the husband should not have another sexual partner without marriage (35). And on the other hand, if a woman is diagnosed with cancer, her husband is allowed to remarry (36), adaptation and its dimensions in Iranian Muslim husbands can have different dimensions. This study aimed to design and validate AQHIMWBC. The final version of the questionnaire included 51 items divided into six dimensions, namely role play, physical disorders, mental tensions associated with economic problems, marital relationships, perceived support, and anticipatory anxiety. In Roy's model, adaptation consisted of four dimensions (physical needs, self-concept, role function, and interdependence) And the set of fears, anxieties, worries are located in the dimension of self-concept due to various causes (29). In the present study, however, Fear of the future and mental tensions related to financial toxicity were allocated to two separate dimensions.

The first dimension obtained in the present study was role play. Accordingly, the husbands tried their best to perform their roles as a husband, father, and worker, so as to manage their wives' disease. In other words, they did their best and tolerated all challenges, pains, and sufferings (16). In contrast, some of the husbands acted passively and considered patient care as being imprisoned in the house, which prevented them from fulfilling their occupational responsibilities (17). However, other studies indicated that husbands considered their supportive role a priority in the treatment of their wives and made genuine attempts to direct their negative emotions through the measures they took. They supported their wives through focusing on their emotions and taking care of them, cooperation in the recovery process, management of the family, and helping them return to normal life (37).

The second dimension of the designed scale in the present research was physical problems such as insomnia, decreased desire to eat, hypertension and heart palpitations. Perndorfer reported the reduced duration and quality of sleep as two major problems among women's husbands, which were related to the fear of cancer recurrence (38).

The third dimension of the scale was related to psychological tensions related to economic problems. Similarly, other studies found that almost one-third of husbands faced financial difficulties and had to work extra hours due to their spouse's illness (39). Also, some of them have suffered financial poisoning, and factors such as the COVID epidemic (40) and sanctions (41) have aggravated this issue, and the cost of treating patients has increased greatly, causing the patient's incomplete treatment to be stopped (42).

The fourth dimension of the scale was related to marital relations. In this context, most of the husbands reported reduced sexual relations and pointed out that women cover their heads and breasts during sex, which reduces the quality of sexual relations, and breast cancer is referred to as relationship cancer (14). This can be attributed mainly to alopecia and mastectomy, which lead to a decrease in the sexual attractiveness of women from the perspective of husbands (43). In this study, some of the patient's husbands left or chose another sexual partner, which is considered unethical and betrayal from the patients' point of view Evidence suggests that women with BC reported a wide range of husbands' behaviors, from fidelity to infidelity, with patient abandonment indicating insufficient husband support for the patient (44).

The fifth dimension is the scale of support perceived by people around, governmental and non-governmental organizations, insurances, therapists in terms of mental, financial and spiritual. Although younger husbands show more tendency to receive support, few studies have paid attention to and evaluated the support of the husband (22), While the all-round support of the husband increases the patient's survival (45).

The sixth dimension of the scale was fear of the future, which mainly resulted from cancer recurrence. This issue has been confirmed in other studies, as well (38). This type of fear can range from mild to severe degrees, and becomes more intense when one's acquaintances are informed about the patient's cancer recurrence (46). Another reason for the fear of the future was financial concerns, which usually increases with the progress of the disease (47), and factors such as sanctions (48) increase their worries.

## Conclusions

Based on the study findings, the designed adaptation scale has an appropriate reliability and validity and can be used amongst Iranian Muslim husbands. One of the main advantages of this scale is that, on the contrary to the previous instruments, it is not one-dimensional and encompasses all the dimensions of adaptation. Additionally, it has been designed on the basis of the target group's experiences. Finally, its reliability and validity have been assessed accurately. Yet, development of a new tool and evaluation of its psychometric properties require continuous processes and, consequently, further attempts are required for improving the designed scale. The researchers also hope to overcome the probable shortcomings related to the scale in future. Performance of confirmatory factor analysis is warranted, as well.

## Limitations

One of the limitations in designing of AQHIMWBC was that due to the corona pandemic, most of the interviews were conducted by telephone and the researcher was introduced to the virtual participants, which may affect the participant's sense of trust, although the researcher did his best before the interview. To create a sense of trust in the participants and by participating in patients' web-based groups and introducing them to prominent group members, this issue was largely resolved.

Considering that most of the participants had been married for more than 20 years and more than 10 years had passed since contracting the disease, it indicates the stability of marriage and the risk of low recurrence in BC survivors, and it reflects long-term compatibility, which is another limitation of the research.

## Data availability statement

The raw data supporting the conclusions of this article will be made available by the authors, without undue reservation.

## Ethics statement

The study received approval by the Ethics Committee of the Vice-chancellor for Research Affairs of Ahvaz Jundishapur University of Medical Sciences (ethics code: IR.AJUMS.REC.1399.099). All participants gave their written consent via web. The patients/participants provided their written informed consent to participate in this study.

## Author contributions

MBBSH and NE wrote the main manuscript text. MBBSH prepared Tables 1–4 and Figure 1. All authors contributed to the article and approved the submitted version.

## References

[1] Ferlay J Soerjomataram I Dikshit R Eser S Mathers C Rebelo M. Cancer incidence and mortality worldwide: sources, methods and major patterns in GLOBOCAN 2012. Int J Cancer. (2015) 136:E359–E86. 10.1002/ijc.29210 25220842

[2] Torre LA Siegel RL Ward EM Jemal A. Global cancer incidence and mortality rates and trends—an update. Cancer Epidemiol Prevent Biomarkers. (2016) 25:16–27. 10.1158/1055-9965.EPI-15-0578 26667886

[3] Siegel RL Miller KD Jemal A. Cancer statistics, 2016. CA Cancer J Clin. (2016) 66:7–30. 10.3322/caac.21332 26742998

[4] Ahmadi A Ramazani R Rezagholi T Yavari P. Incidence pattern and spatial analysis of breast cancer in Iranian women: geographical information system applications. Eastern Mediterranean Health J. (2018) 24:360–7. 10.26719/2018.24.4.360 29972230

[5] Rafiemanesh H Salehiniya H Lotfi Z. Breast cancer in Iranian woman: incidence by age group, morphology and trends. Asian Pacific J Cancer Prevent. (2016) 17:1393–7. 10.7314/APJCP.2016.17.3.1393 27039778

[6] Greenlee H DuPont-Reyes MJ Balneaves LG Carlson LE Cohen MR Deng G. Clinical practice guidelines on the evidence-based use of integrative therapies during and after breast cancer treatment. CA Cancer J Clin. (2017) 67:194–232. 10.3322/caac.21397 28436999 PMC5892208

[7] Moo TA Sanford R Dang C Morrow M. Overview of breast cancer therapy. PET Clin. (2018) 13:339–54. 10.1016/j.cpet.2018.02.006 30100074 PMC6092031

[8] Dixson BJ Duncan M Dixson AF. The role of breast size and areolar pigmentation in perceptions of women's sexual attractiveness, reproductive health, sexual maturity, maternal nurturing abilities, and age. Arch Sex Behav. (2015) 44:1685–95. 10.1007/s10508-015-0516-2 25828990

[9] Dominic NA Arasoo VJT Botross NP Riad A Biding C Ramadas A. Changes in health-related quality of life and psychosocial wellbeing of breast cancer survivors: findings from a group-based intervention program in Malaysia. Asian Pacific J Cancer Prevent. (2018) 19:1809. 10.22034/APJCP.2018.19.7.1809 30049192 PMC6165648

[10] Fouladi N Pourfarzi F Dolattorkpour N Alimohammadi S Mehrara E. Sexual life after mastectomy in breast cancer survivors: a qualitative study. Psycho Oncol. (2018) 27:434–41. 10.1002/pon.4479 28618128

[11] Nakaya N Saito-Nakaya K Bidstrup PE Dalton SO Frederiksen K Steding-Jessen M. Increased risk of severe depression in male partners of women with breast cancer. Cancer. (2010) 116:5527–34. 10.1002/cncr.25534 20878654

[12] Kaewkerd O Chaiyasit Y Vibulchai S Kenthongdee W Sirisawat M Panput A. Key factors of family adaptation to the illness of family members: an integrative review. Bangkok Med J. (2020) 16:95. 10.31524/bkkmedj.2020.13.002

[13] Borstelmann NA Rosenberg SM Ruddy KJ Tamimi RM Gelber S Schapira L. Partner support and anxiety in young women with breast cancer. Psycho Oncol. (2015) 24:1679–85. 10.1002/pon.3780 25765893

[14] Fang SY Chang HT Shu BC. The moderating effect of perceived partner empathy on body image and depression among breast cancer survivors. Psycho Oncol. (2015) 24:1815–22. 10.1002/pon.3868 26110591

[15] Fang S-Y Lin Y-C Chen T-C Lin C-Y. Impact of marital coping on the relationship between body image and sexuality among breast cancer survivors. Supportive Care in Cancer. (2015) 23:2551–9. 10.1007/s00520-015-2612-1 25617071

[16] Younes Barani Z Rahnama M Naderifar M Badakhsh M Noorisanchooli H. Experiences of spouses of women with breast cancer: a content analysis. Asian Pac J Cancer Prev. (2019) 20:3167–72. 10.31557/APJCP.2019.20.10.3167 31653169 PMC6982647

[17] Shannon CS. ‘I was trapped at home': men's experiences with leisure while giving care to partners during a breast cancer experience. Leisure Sci. (2015) 37:125–41. 10.1080/01490400.2014.973128

[18] Lazarus RS Folkman S. Stress, Appraisal, and Coping. Berlin: Springer (1984).

[19] Endler NS Parker J. Coping Inventory for Stressful Situations: Multi-Health Systems Incorporated (1990).

[20] Amirkhan JH. A factor analytically derived measure of coping: the coping strategy indicator. J Pers Soc Psychol. (1990) 59:1066. 10.1037/0022-3514.59.5.1066

[21] Franco LR Garcia FC Picabia AB. Assessment of chronic pain coping strategies. Actas Esp Psiquiatr. (2004) 32:82–91. 15042468

[22] Flink IK Thomtén J Engman L Hedström S Linton SJ. Coping with painful sex: development and initial validation of the CHAMP sexual pain coping scale. Scandinavian J Pain. (2015) 9:74–80. 10.1016/j.sjpain.2015.05.002 29911654

[23] Moorey S Frampton M Greer S. The cancer coping questionnaire: a self-rating scale for measuring the impact of adjuvant psychological therapy on coping behaviour. Psycho-Oncology. (2003) 12:331–44. 10.1002/pon.646 12748971

[24] Carver CS Scheier MF Weintraub JK. Assessing coping strategies: a theoretically based approach. J Pers Soc Psychol. (1989) 56:267. 10.1037/0022-3514.56.2.267 2926629

[25] Doumit MA Huijer HA-S Kelley JH El Saghir N Nassar N. Coping with breast cancer: a phenomenological study. Cancer Nurs. (2010) 33:E33–E9. 10.1097/NCC.0b013e3181c5d70f 20142735

[26] Mir-Hosseini Z. The politics of divorce laws in iran: ideology versus practice. Interpreting Divorce Laws Islam. (2012) 65–83.

[27] Bakhshizadeh M. An Analysis of Possibility of Fulfilling Gender Equality Within the Legal System of the Islamic Republic of Iran (IRI). Law, Religion and Tradition. Berlin: Springer (2018) p. 43–70. 10.1007/978-3-319-96749-3_3

[28] Yusoff MSB Arifin WN Hadie SNH. ABC of questionnaire development and validation for survey research. Edu Med J. (2021) 13. 10.21315/eimj2021.13.1.10

[29] Harris R. Sister Callista Roy: Adaptation Model. Nursing Theorists and Their Work E-Book. (2021). p. 247.

[30] Elo S Kyngäs H. The qualitative content analysis process. J Adv Nurs. (2008) 62:107–15. 10.1111/j.1365-2648.2007.04569.x 18352969

[31] Banning M Hafeez H Faisal S Hassan M Zafar A. The impact of culture and sociological and psychological issues on Muslim patients with breast cancer in Pakistan. Cancer Nurs. (2009) 32:317–24. 10.1097/NCC.0b013e31819b240f 19444089

[32] Ghaderi I Kaviani A Fakhrejahani E Mehrdad N Hazar N Karbakhsh M. Religious, cultural, and social beliefs of iranian rural women about breast cancer: a qualitative study. Arch Breast Cancer. (2014) 1:25–31.

[33] Kayser K Cheung PK Rao N Chan YCL Chan Y Lo PH. The influence of culture on couples coping with breast cancer: a comparative analysis of couples from China, India, and the United States. J Psychosoc Oncol. (2014) 32:264–88. 10.1080/07347332.2014.897292 24611914

[34] Ghiabi M Maarefvand M Bahari H Alavi Z. Islam and cannabis: legalisation and religious debate in Iran. Int J Drug Policy. (2018) 56:121–7. 10.1016/j.drugpo.2018.03.009 29635140 PMC6153265

[35] Afary J. Sexual Politics in Modern Iran. Cambridge: Cambridge University Press (2009). 10.1017/CBO9780511815249

[36] Johnson V. A note on the operation of the dissolution of muslim marriages act, 1939. J Divorce Remarriage. (2011) 52:94–108. 10.1080/10502556.2011.546227

[37] Zierkiewicz E Mazurek E. Couples dealing with breast cancer—the role of husbands in supporting their wives. Studia Humanistyczne. (2015) 14:95–116. 10.7494/human.2015.14.2.95

[38] Perndorfer C. Fear of Cancer Recurrence and Sleep in Couples Coping with Early-stage Breast Cancer. Newark, Delaware: University of Delaware (2019). 10.1093/abm/kaac018 PMC9635995 35551585

[39] Veenstra CM Wallner LP Jagsi R Abrahamse P Griggs JJ Bradley CJ. Long-term economic and employment outcomes among partners of women with early-stage breast cancer. J Oncol Practice. (2017) 13:e916–e26. 10.1200/JOP.2017.023606 28880714 PMC5684882

[40] Thom B Benedict C Friedman DN Watson SE Zeitler MS Chino F. Economic distress, financial toxicity, and medical cost-coping in young adult cancer survivors during the COVID-19 pandemic: findings from an online sample. Cancer. (2021) 127:4481–91. 10.1002/cncr.33823 34351638 PMC8426858

[41] Shahabi S Fazlalizadeh H Stedman J Chuang L Shariftabrizi A Ram R. The impact of international economic sanctions on Iranian cancer healthcare. Health Policy. (2015) 119:1309–18. 10.1016/j.healthpol.2015.08.012 26344426

[42] Abdoli A. Iran, sanctions, and the COVID-19 crisis. J Med Econ. (2020) 23:1461–5. 10.1080/13696998.2020.1856855 33249954

[43] Ghizzani A Bruni S Luisi S. The sex life of women surviving breast cancer. Gynecol Endocrinol. (2018) 34:821–5. 10.1080/09513590.2018.1467401 29703097

[44] Nouri Sanchuli H Rahnama M Shahdadi H Poudineh Moghaddam M. From love and fidelity to infidelity-individual experiences of women with breast cancer regarding relationships with their spouses. Asian Pacific J Cancer Prevent. (2017) 18:2861–6. 10.22034/APJCP.2017.18.10.2861 29072437 PMC5747415

[45] Suwankhong D Liamputtong P. Social support and women living with breast cancer in the south of Thailand. J Nursing Scholarship. (2016) 48:39–47. 10.1111/jnu.12179 26580861

[46] Butow P Sharpe L Thewes B Turner J Gilchrist J Beith J. Fear of cancer recurrence: a practical guide for clinicians. Oncology. (2018) 32:32–8. 29447419

[47] Rosenzweig M West M Matthews J Stokan M Kook Y Gallups S. Financial toxicity among women with metastatic breast cancer. Oncol Nursing Forum. (2019) 46:1. 30547962 10.1188/19.ONF.83-91

[48] Akbarialiabad H Rastegar A Bastani B. How sanctions have impacted Iranian healthcare sector: a brief review. Arch Iran Med. (2021) 24:58. 10.34172/aim.2021.09 33588569

